# Identification of mini‐chromosome maintenance 8 as a potential prognostic marker and its effects on proliferation and apoptosis in gastric cancer

**DOI:** 10.1111/jcmm.16062

**Published:** 2020-11-06

**Authors:** Bin Huang, Minghe Lin, Lisha Lu, Wujin Chen, Jingzhuang Tan, Jinyan Zhao, Zhiyun Cao, Xiaoqin Zhu, Jiumao Lin

**Affiliations:** ^1^ Academy of Integrative Medicine Fujian University of Traditional Chinese Medicine Fuzhou China; ^2^ Fujian Key Laboratory of Integrative Medicine on Geriatrics Fujian University of Traditional Chinese Medicine Fuzhou China; ^3^ Department of Oncology Affiliated People’s Hospital of Fujian University of Traditional Chinese Medicine Fuzhou China

**Keywords:** apoptosis, gastric cancer, MCM8, proliferation, survival

## Abstract

Mini‐chromosome maintenance (MCM) proteins play important roles in initiating eukaryotic genome replication. The MCM family of proteins includes several members associated with the development and progression of certain cancers. We performed online data mining to assess the expression of MCMs in gastric cancer (GC) and the correlation between their expression and survival in patients with GC. Notably, MCM8 expression was undoubtedly up‐regulated in GC, and higher expression correlated with shorter overall survival (OS) and progression‐free survival (PFS) in patients with GC. However, the role of MCM8 in GC has not been previously explored. Our in vitro experiments revealed that MCM8 knockdown inhibited cell growth and metastasis. Moreover, MCM8 knockdown induced apoptosis. Mechanistically, the expression levels of Bax and cleaved caspase‐3 were increased, whereas Bcl‐2 expression decreased. Additionally, we demonstrated that MCM8 knockdown suppressed tumorigenesis in vivo. Overall, these results suggest that MCM8 plays a significant role in GC progression.

## 1 INTRODUCTION

Stomach cancer, also known as gastric cancer (GC), is the fifth most common type of cancer, presenting the third‐highest mortality worldwide and accounting for 1.03 million new cases and over 783 000 deaths in 2018.^1^ Surgery combined with chemotherapy and radiotherapy are major treatment strategies for GC. However, GC is virtually untreatable unless detected at an early stage. The clinical translation of molecular‐guided targeted therapy is hampered by several challenges.^2^ Therefore, further exploration of molecular mechanisms underlying GC is essential to determine innovative and useful therapeutic targets.

The mini‐chromosome maintenance (MCM) family plays an important role in initiating eukaryotic genome replication.^3^ Moreover, MCM family proteins are involved in replication, elongation, cohesion, condensation, transcription and recombination of DNA molecules; these roles were first identified in budding yeast, *Saccharomyces cerevisiae*.^4^ This protein family includes at least 10 proteins. MCM1 regulates cell proliferation, apoptosis and differentiation.^5^ Additionally, MCM proteins 2‐7 are important and play a role in DNA replication and elongation,^4^, ^6^, ^7^, ^8^ with this complex displaying helicase activity in vitro.^9^, ^10^ Subsequently, MCM8 and MCM9 were discovered, similar to other members of the MCM2, MCM3, MCM4, MCM5, MCM6, MCM7 proteins^3^ and are crucial for DNA pre‐replication and initiation of the S phase.^11^, ^12^ In addition, they facilitate homologous recombination repair as a heterohexameric MCM8 and MCM9 complex at DNA damage sites.^11^, ^13^, ^14^ MCM10 is an additional essential protein for the initiation of DNA synthesis.^15^, ^16^

Recently, increasing evidence has suggested that MCMs are up‐regulated in multiple malignancies including cervical cancer,^17^ breast cancer,^18^ oesophageal squamous cell cancer,^19^ chronic myelogenous leukaemia,^20^ human gliomas and non–small‐cell lung cancer.^21^, ^22^, ^23^, ^24^ However, there is less evidence demonstrating the relationship between MCM family proteins and GC. Therefore, we assessed the mRNA expression of MCMs in GC when compared with normal adjacent parental tissues by GEPIA (Gene Expression Profiling Interactive Analysis). Additionally, we analysed the relationship between MCM expression and the progression and prognosis of GC using the Kaplan‐Meier plotter analysis. We observed that MCM2, MCM5 and MCM8 expression was up‐regulated in GC samples when compared with adjacent normal parental samples and correlated with a poor prognosis. Meanwhile, there have been several articles reveal that MCM2 and MCM5 may serve as prognostic indicators of patients with gastric cancer, but there is no such article on MCM8. Moreover, there is no in vitro or in vivo study on the function of MCM8 in gastric cancer.^25^, ^26^, ^27^ We performed further analyses on MCM8. We detected the mRNA expression of MCM8 and its association with overall survival (OS) and progression‐free survival (PFS) in different GC databases. Furthermore, functional assays indicated that MCM8 knockdown significantly inhibited cell growth and metastasis, but induced cell apoptosis. Intrinsic and extrinsic pathways are two major apoptotic pathways. The intrinsic pathway, also called the mitochondrial pathway, is attributed to the essential involvement of mitochondria.^28^ The intrinsic pathway of apoptosis is regulated by a family of proteins called the Bcl‐2 family. Some of these proteins (such as Bad, Bax or Bid) are pro‐apoptotic, while others (such as Bcl‐2 and Bcl‐XL) are anti‐apoptotic. The balance between pro‐ and anti‐apoptotic Bcl‐2 proteins determines the sensitivity of cells to apoptotic stimuli.^29^ Cleaved caspase‐3 is an important indicator of apoptosis.^28^ Thus, we further determined the expression levels of Bcl‐2, Bax and cleaved caspase‐3 to uncover the underlying mechanism. In addition, our study showed that MCM8 knockdown suppressed the development and progression of cancer in vivo. Therefore, MCM8 may be a potential target for GC treatment.

## 2 MATERIALS AND METHODS

### 2.1 Bioinformatics and survival analysis

MCM mRNA expression in GC cancers vs normal tissues was analysed using GEPIA (http://gepia.cancer-pku.cn/) and Oncomine (http://www.oncomine.org). For the survival analysis, Kaplan‐Meier plotter (http://kmplot.com/analysis/)30 was used to assess the OS and PFS in patients. Based on the median MCM8 expression level, patients with GC were classified into low and high expression groups, analysing the relationship using the log‐rank test.

### 2.2 Cell lines

The GC cell lines, AGS and HGC27, were obtained from ATCC (Manassas, VA, USA). AGS and HGC27 cells were cultured in RPMI‐1640 medium (Gibco; Carlsbad, CA, USA) containing 10% (v/v) foetal bovine serum (FBS; Gibco), 100 U/mL penicillin and 100 µg/mL streptomycin (Hyclone; Logan, UT, USA) at 37°C in a humidified atmosphere containing 5% CO_2_.

### 2.3 Plasmid construction and lentiviral transduction

Three independent shRNAs targeting MCM8 and a control shRNA were designed by Shanghai HanBio Company (Shanghai, China). The shRNAs were cloned into the lentivirus‐based vector pHBLV‐U6‐MCS‐PGK‐PURO. The targeting sequence of the shRNAs and primers for plasmid construction is presented in Table S1. The lentiviruses carrying MCM8 shRNA or control shRNA were purchased from Shanghai HanBio Biotechnology. Briefly, cells were seeded in 24‐well plates and infected with shRNA or sh‐Ctrl lentivirus at a multiplicity of infection (MOI) of 30 at 37°C, according to instructions provided. Two days after infection, cells were maintained in 1.0 µg/mL of puromycin (Sigma, San Francisco, CA, USA) for 7 days. Then, stable MCM8‐knockdown cells and control cells were used in the following experiments.

### 2.4 Quantitative reverse transcription polymerase chain reaction (RT‐qPCR)

Total RNA was extracted from cells using RNAiso Plus reagent (Takara; Dalian, Liaoning, China) and reverse‐transcribed (oligo) into cDNA with the PrimeScript RT kit (Takara). Next, gene expression was detected as mRNA levels by qPCR with the ABI 7500 Fast Real‐Time PCR System (Applied Biosystems; Carlsbad, CA, USA), and GAPDH was used as the input reference. The thermocycling conditions were as follows: 95°C for 5 minutes, followed by 45 cycles of 95°C for 20 seconds, 58°C for 20 seconds and 72°C for 20 seconds. Each detection was performed in triplicate. The primers used are shown in Table 1. Relative mRNA was calculated using the formula 2‐ÄÄCT (with CT being the cycle threshold), in which ÄCT = [CT (target gene) ‐ CT (GAPDH)], as described previously.^30^

**TABLE 1:** Primers for qPCR

MCM8 forward primer	5′‐TCTCCTCTCACAGTTACGATGG‐3′
MCM8 reverse primer	5′‐TGCTTACACCCATCCTCAGAAC‐3′
Bcl‐2 forward primer	5′‐ TCGCCCTGTGGATGACTGAGT ‐3′
Bcl‐2 reverse primer	5′‐GCCAGGAGAAATCAAACAGAGGC‐3′
Bax forward primer	5′‐TCAGGATGCGTCCACCAAGAAG‐3′
Bax reverse primer	5′‐TGTGTCCACGGCGGCAATCATC‐3′

### 2.5 Western blot analysis

Briefly, transfected cells were lysed with lysis buffer including cocktails and phenylmethylsulfonyl fluoride (PMSF). The protein concentration in the whole‐cell extracts was measured using the BCA Protein Assay Kit (Pierce). Identical amounts of protein were loaded in SDS‐PAGE gel and then transferred onto PVDF membranes, blocked with Blocking Buffer (Thermo) at 28°C for 1 hour and probed with primary antibodies (dilution, 1:1000) at 4°C overnight. All antibodies, except MCM8, were obtained from Cell Signaling Technology (CST; Beverly, MA, USA); MCM8 was purchased from Thermo Fisher Scientific (Carlsbad, CA, USA. Cat#PA5‐41325). Then, membranes were washed with the wash buffer and incubated with goat anti‐rabbit HRP (horseradish peroxidase) secondary antibodies (1:5000; CST, #7074) at 28°C. After incubation with the chemiluminescence detection reagent, the bands were visualized and analysed with the ImageLab software. The protein level of β‐actin was used as a loading control.

### 2.6 CCK‐8 assay

Cell proliferation was determined with the Cell Counting Kit‐8 (CCK‐8; Dojindo; Japan). In brief, transfected cells were seeded onto 96‐well plates (2000 cells/well in 100 μL of medium) and incubated at 37°C in a humidified atmosphere containing 5% CO_2_. CCK‐8 (10 μL) was added to the plates and incubated for an additional 2 hours at 37°C. Finally, the OD was measured at 450 nm using the Infinite 200 Pro microplate reader (Tecan; Männedorf, Switzerland) at each indicated time‐point.

### 2.7 Colony formation assay

In brief, transduced cells were reseeded into 6‐well plates (1000 cells/well) and incubated for 10‐12 days at 37°C in a humidified atmosphere containing 5% CO_2_. RPMI‐1640 medium was replaced every 2‐3 days; then, colonies were washed with PBS, fixed them with 4% formaldehyde and then stained with crystal violet. Finally, the total number of colonies was counted, and images were obtained.

### 2.8 Cell cycle analysis

FxCycle PI/RNase Staining Solution was applied to transfected cells at 28°C, according to the manufacturer's instructions. Cell cycle phases were measured using FACS Caliber (Becton‐Dickinson; San Jose, CA, USA). The percentage of DNA in different phases was analysed using ModfitLT version 3.0 (Verity Software House, Topsham, ME, USA).

### 2.9 Apoptosis analysis

Apoptotic cells were stained with annexin V‐fluorescein isothiocyanate (FITC) (Cat: A211‐02, Vazyme, Nanjing, China) and 7‐amino‐actinomycin (7‐AAD) (Cat: A213‐01, Vazyme) in the dark for 10 minutes at 28°C, according to the manufacturer's instructions. After staining, the samples were immediately analysed using a flow cytometer (FACS Caliber, Becton‐Dickinson).

### 2.10 Transwell assay

Transwell plates (Corning, Corning, NY, USA) with 8‐μm‐pore size membranes were used to perform Transwell migration and Matrigel invasion assays. In brief, 3 × 10^4^ cells were suspended using 100 μL FBS‐free RPMI‐1640 medium and seeded into the upper chambers of transwell plates. The lower chambers contained 500 μL RPMI‐1640 medium supplied with 5% FBS. After a 24‐hour incubation period at 37°C, 0.5% toluidine blue was used to stain migrated cells, and the number of migrated cells was counted in three random fields. The membranes of the upper chambers were pre‐coated with 8‐fold diluted Matrigel (BD Biosciences, Sparks, MD) before use in the Matrigel invasion assay.

### 2.11 Tumour xenograft

Six‐ to‐eight‐week‐old female nude mice were purchased from Charles River (Beijing, China). HGC27 cells (4 × 10^6^), with or without MCM8 knockdown, were subcutaneously injected into the right‐side dorsal flank of each mouse. Tumours were isolated on day 38, with the length (a) and width (b) of tumours recorded every 4 days. The tumour volume was calculated using the following formula, V = ab^2^/2 (cm^3^). Additionally, the tumours were imaged on day 38 after injection.

### 2.12 Statistical analysis

Data were analysed using SPSS 20.0 (IBM Corp., Armonk, NY, USA). The qPCR results were evaluated with one‐way ANOVA, and other results were analysed using Student's *t* test, presenting the means ± standard deviation (SD) obtained from three independent experiments. A *P*‐value of <.05 was considered statistically significant.

## 3 RESULTS

### 3.1 mRNA expression of MCMs in GC samples

**FIGURE 1:**
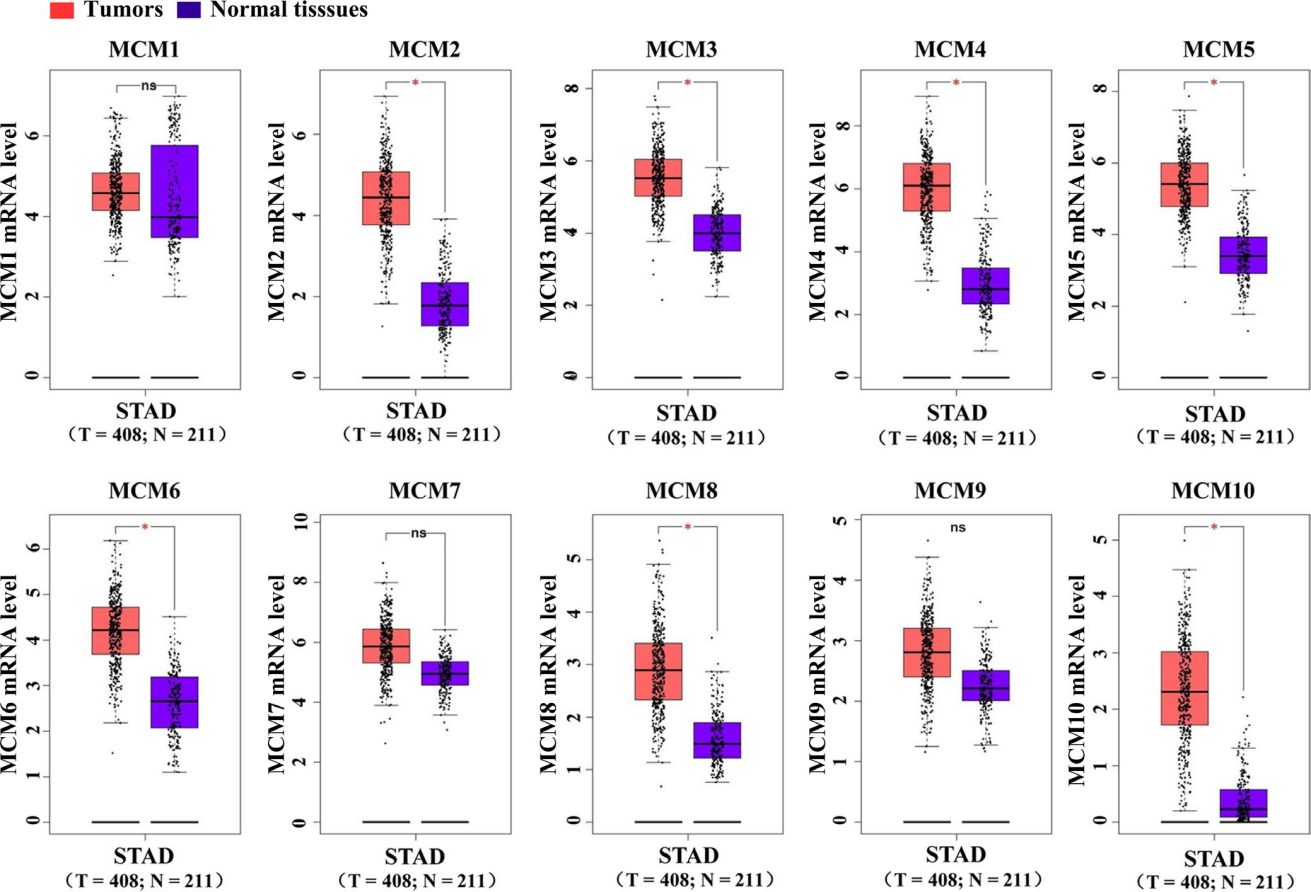
The mRNA expression of MCMs in GC tissues. The mRNA expression of MCMs in GC and normal tissues was compared based on the GEPIA database. *P < .05, tumour vs normal tissues. MCM, mini‐chromosome maintenance; GC, gastric cancer

The mRNA expression levels of MCMs in GC and normal tissues were compared based on data from GEPIA. As shown in Figure 1, mRNA levels of MCM2, MCM3, MCM4, MCM5, MCM6, MCM8 and MCM10 were significantly up‐regulated in GC tissues when compared with normal tissues. However, mRNA levels of MCM1, MCM7 and MCM9 did not significantly differ between GC and normal tissues.

### 3.2 Relationship between MCM expression and prognosis in patients with GC

**FIGURE 2:**
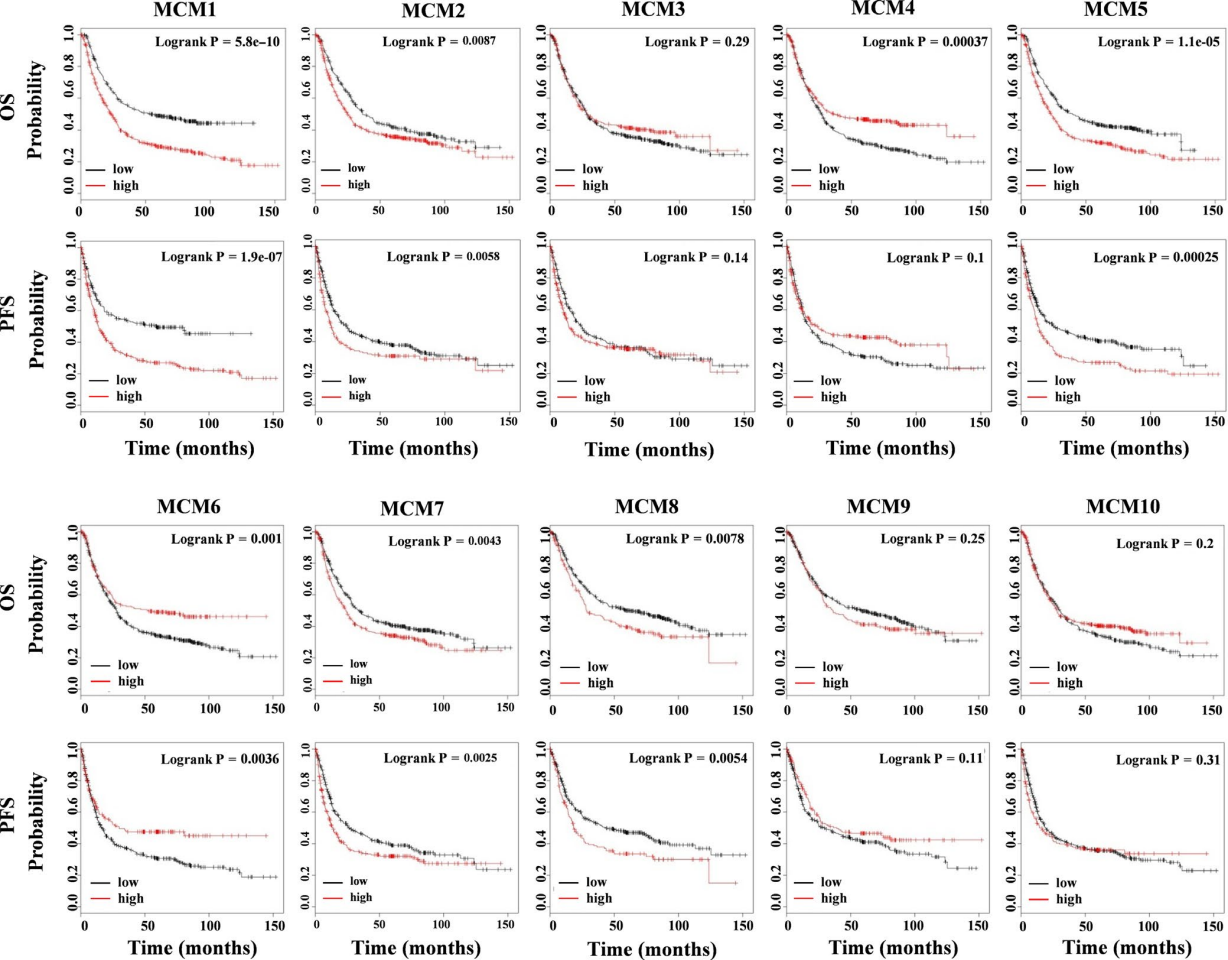
The relationship between MCM expression and poor prognosis in patients with GC. Survival analysis was based on MCM expression in patients with GC from the public clinical microarray data set using the Kaplan‐Meier plotter analysis (*P < .05). Survival was analysed using a log‐rank test. MCM, mini‐chromosome maintenance; GC, gastric cancer

In patients with GC patients, the association of MCMs with OS and PFS was analysed by the Kaplan‐Meier method. As shown in Figure 2, the survival curves for MCM1, MCM2, MCM5, MCM7 and MCM8 indicated that patients with high expression levels may present a shorter OS and PFS than those with low expression levels (*P* < .05). Conversely, patients with high MCM4 or MCM6 expression exhibited a longer OS (*P* < .05). Moreover, high MCM6 expression may indicate a higher PFS (*P* < .05). In addition, expression levels of MCM3, MCM9 and MCM10 did not significantly affect both OS and PFS.

### 3.3 MCM8 is highly expressed in GC tissues and is associated with poor prognosis in patients with GC

**FIGURE 3:**
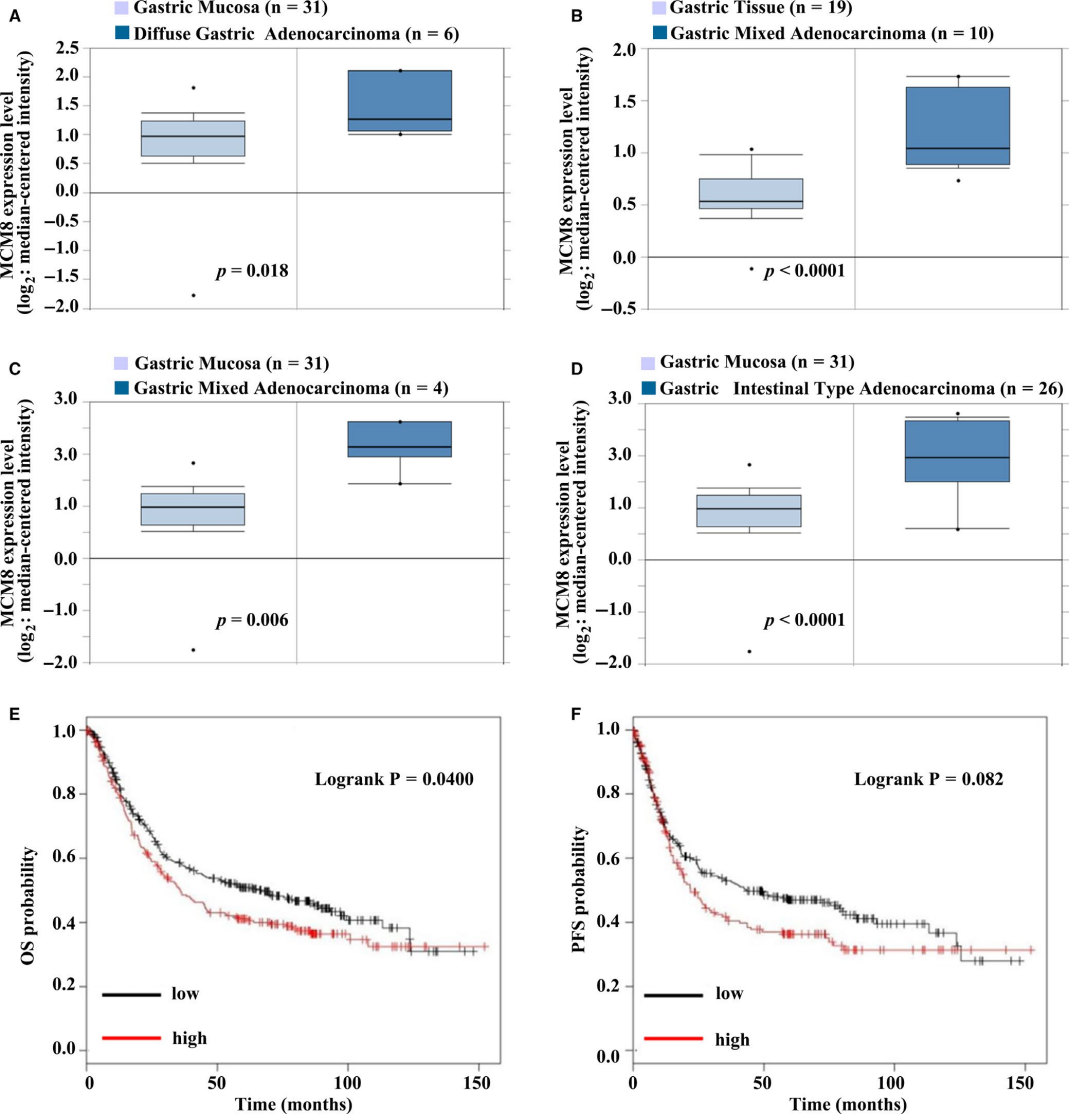
mRNA expression of MCM8 in GC tissues and the relationship with poor prognosis in patients with GC. A‐D, MCM8 expression in different types of GC and normal tissues was compared based on the Oncomine database. A, Reporter: 224320_s_at. B, Reporter: ILMN_2047124. C, Reporter: 224320_s_at. D, Reporter: 224320_s_at. E‐F, The association of MCM8 with overall survival (left) in 631 GC patients and progression‐free survival (right) in 522 GC patients was analysed using the Kaplan‐Meier method (*P < .05). Survival was analysed using a log‐rank test

Based on the above data, it appeared that MCM2, MCM5 and MCM8 may be potentially useful biomarkers. However, there have been several articles reveal that MCM2 and MCM5 may serve as prognostic indicators of patients with gastric cancer, but there is no such article on MCM8. Moreover, there is no in vitro or in vivo study on the function of MCM8 in gastric cancer. So, we focused our attention on MCM8. In GC and normal tissues, the expression of MCM8 was compared based on data from Oncomine. In GC tissues, MCM8 mRNA levels were approximately increased by 1.5‐ to 2‐fold when compared with levels observed in normal tissues (Figure 3A‐D). The association of MCM8 with OS (in 631 patients with GC) and PFS (in 522 patients with GC) was analysed using the Kaplan‐Meier method. Based on the classified groups, the OS curve indicated that patients with low MCM8 expression levels demonstrated a higher survival rate than those with high MCM8 expression (*P* < .05, Figure 3E); thus, the up‐regulation of MCM8 was associated with poor prognosis in patients with GC. This finding suggests that MCM8 might act as a prognostic biomarker. However, no significant difference was observed between the low and high expression groups for PFS (log‐rank *P* = .082) (Figure 3F).

### 3.4 MCM8 knockdown inhibits the growth of GC cells

**FIGURE 4:**
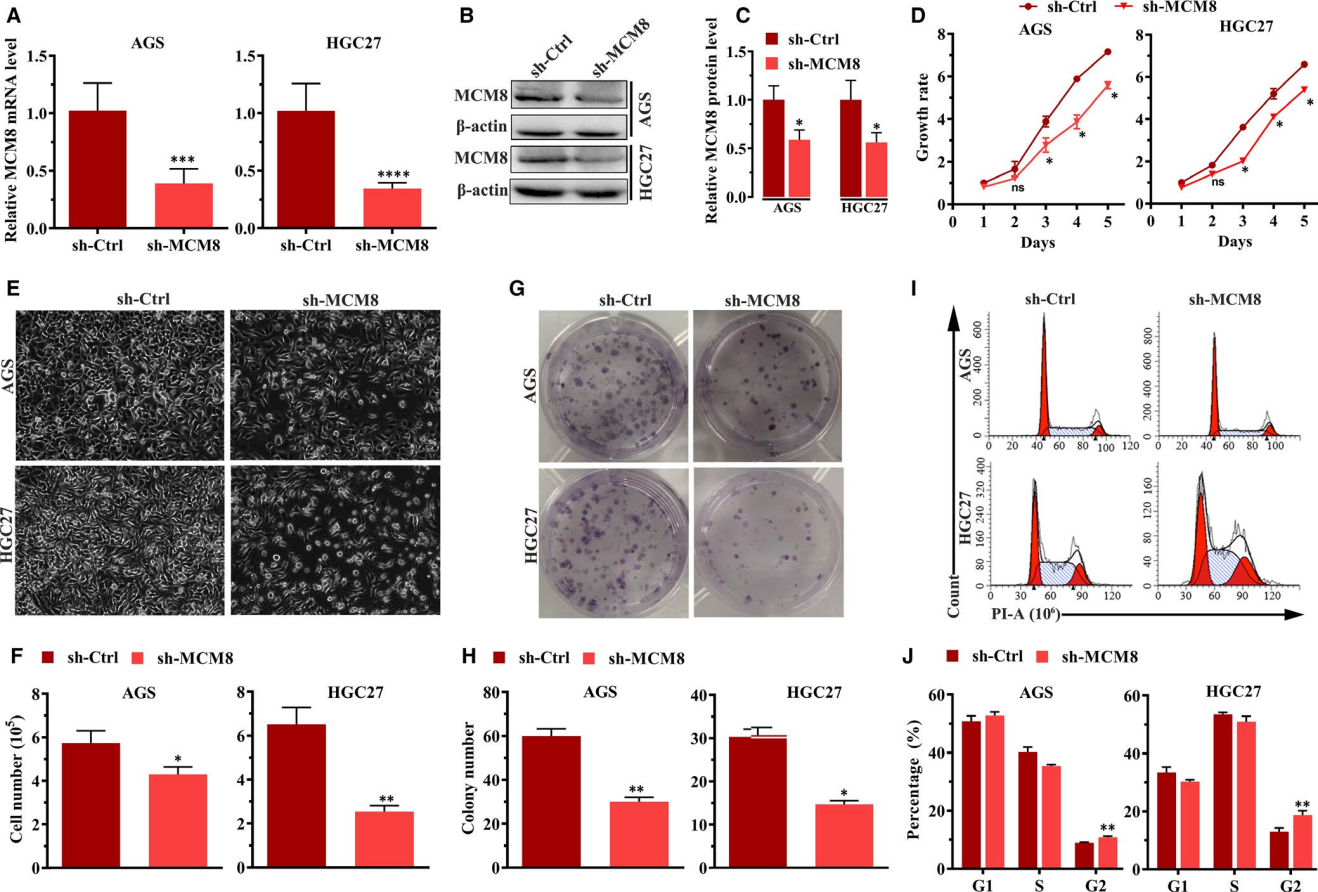
MCM8 knockdown inhibits cell growth. A, MCM8 mRNA levels in AGS and HGC27 cells were detected by RT‐qPCR. ***P < .001, ****P < .0001. B, Analysis of Western blotting showing that protein levels of MCM8 are reduced after MCM8 knockdown. C, Semi‐quantification of Western blotting. The integrated band density was determined using the ImageLab Software, and β‐actin was used as the reference. *P < .05. D, MTT assay showing that MCM8 knockdown inhibits cell viability. ns: not significant. *P < .05. E, Morphological changes in AGS and HGC cells after infection with sh‐MCM8 vs sh‐Ctrl lentiviruses for 72 h. Cell morphology was observed under a phase‐contrast microscope. Images were obtained at a magnification of 200×. F, MCM8 knockdown inhibits cell growth. *P < .05, **P < .01. G, Representative images of colony formation assay. H, MCM8 knockdown reduces the number of colonies. *P < .05, **P < .01. I, Representative images of cell cycle assay by flow cytometry. J, MCM8 knockdown induces arrest at the G2/M phase. **P < .01. RT‐qPCR, quantitative reverse transcription polymerase chain reaction

Three independent shRNAs targeting MCM8 were designed, and knockdown efficiencies were determined using RT‐qPCR. We selected sh‐MCM8‐1, presenting the highest efficiency (Figure S1), to package the lentivirus. Then, we used the lentivirus to infect AGS and HGC27 cells to construct stable MCM8‐knockdown cells. Both mRNA and protein levels of MCM8 were significantly lower than control cells (Figure 4A‐C). Next, we used stable cells (AGS/sh‐Ctrl and AGS/sh‐MCM8, HCG27/sh‐Ctrl and HGC27/sh‐MCM8) to evaluate the effects of MCM8 on cellular functions. As shown in Figure 4 D‐4F, MCM8 knockdown reduced the number and viability of AGS and HGC27 cells. Moreover, MCM8 knockdown inhibited the formation of colonies (Figure 4G). The colony number reduced from 60 ± 5 to 30 ± 3 (*P* = .0014) and from 30 ± 3 to 14 ± 1 (*P* = .0014) in AGS and HGC27 cells, respectively (Figure 4H). To further elucidate the possible mechanism underlying cellular growth inhibition induced by MCM8 knockdown, the cell cycle assay was evaluated by flow cytometry. As shown in Figure 4I, MCM8 knockdown significantly increased the percentage of cells in the G2/M phase. The percentages were increased from 8.9 ± 0.2% to 10.8 ± 0.4% (*P* = .0022) in AGS cells and from 13.0 ± 1.0% to 18.7 ± 1.6% (*P* = .0077) in HGC27 cells (Figure 4J). These results suggested that MCM8 knockdown may inhibit proliferation by inducing G2/M phase arrest.

### 3.5 MCM8 knockdown induces apoptosis in GC cells

**FIGURE 5:**
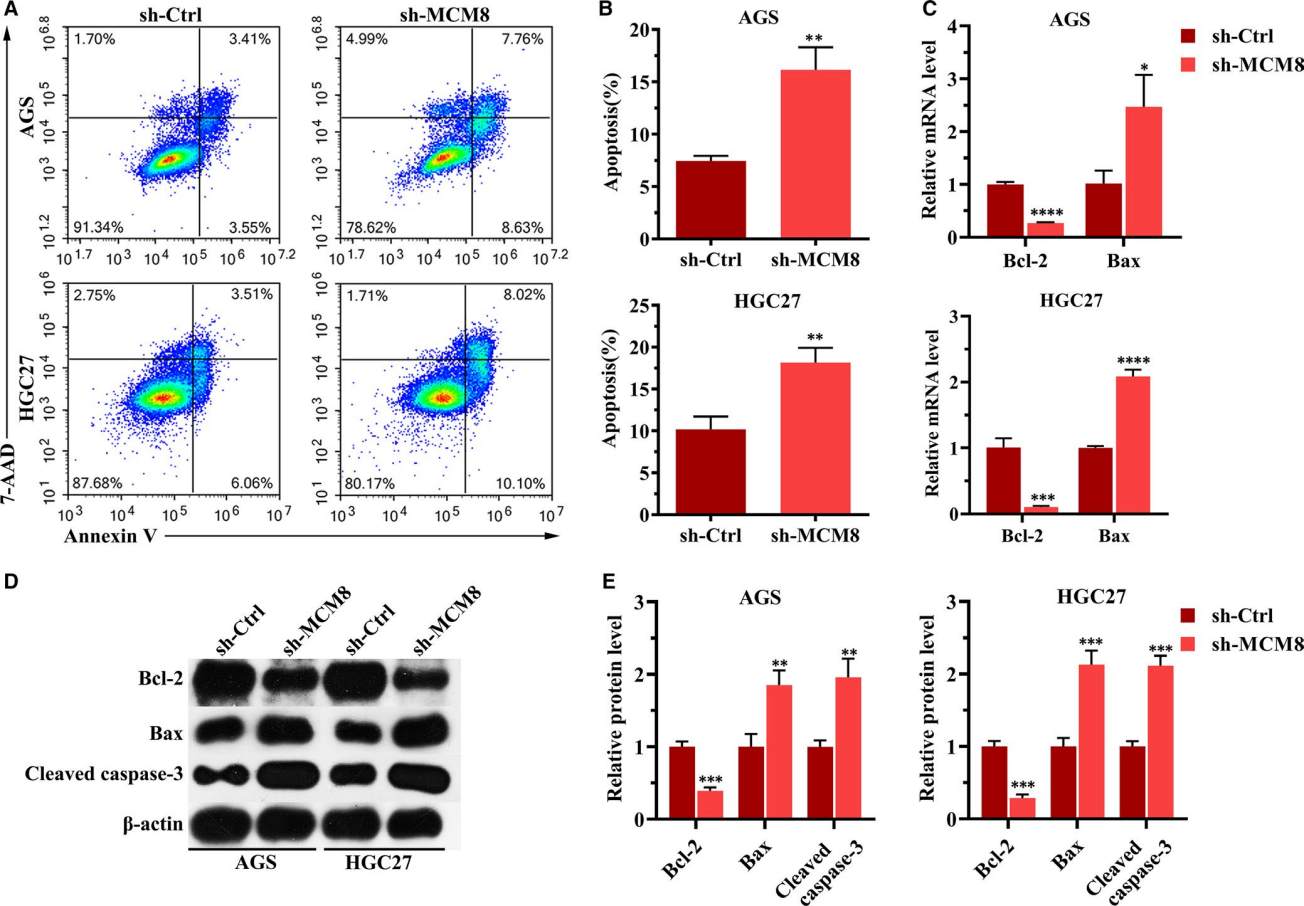
MCM8 knockdown induces cell apoptosis. A, Detection of apoptotic cells stained with Annexin V‐FITC and 7‐AAD by flow cytometry. B, MCM8 knockdown promotes cell apoptosis. **P < .01. C, Determination of Bcl‐2 and Bax mRNA levels using RT‐qPCR. GAPDH was used as a reference. *P < .05, ***P < .001, ***P < .0001. D, Protein levels of Bcl‐2, Bax and cleaved caspase‐3 were determined using Western blotting. β‐actin was used as the loading control. E, Semi‐quantification of Western blotting. The integrated band density was determined using ImageLab Software, and β‐actin was used as the reference. **P < .01, ***P < .001. RT‐qPCR, quantitative reverse transcription polymerase chain reaction

The effect of MCM8 on cell apoptosis was investigated. Annexin V‐FITC and 7‐AAD were used to stain apoptotic cells. As shown in Figure 5A,B, MCM8 knockdown significantly increased the percentage of apoptotic cells, from 7.5 ± 0.5% to 16.2 ± 2.2% for AGS cells and from 10.2 ± 1.5% to 18.2 ± 1.7% for HGC27. Mechanistically, both mRNA expression and protein levels of Bcl‐2 were down‐regulated following MCM8 knockdown, whereas the expression level of Bax was up‐regulated (Figure 5C‐E). The increased protein level of cleaved caspase‐3 indicated that MCM8 knockdown promotes apoptosis (Figure 5D,E). Collectively, MCM8 knockdown may induce apoptosis by regulating the expressions of Bcl‐2 and Bax.

#### 3.5.1 MCM8 knockdown suppresses cell migration and invasion

**FIGURE 6:**
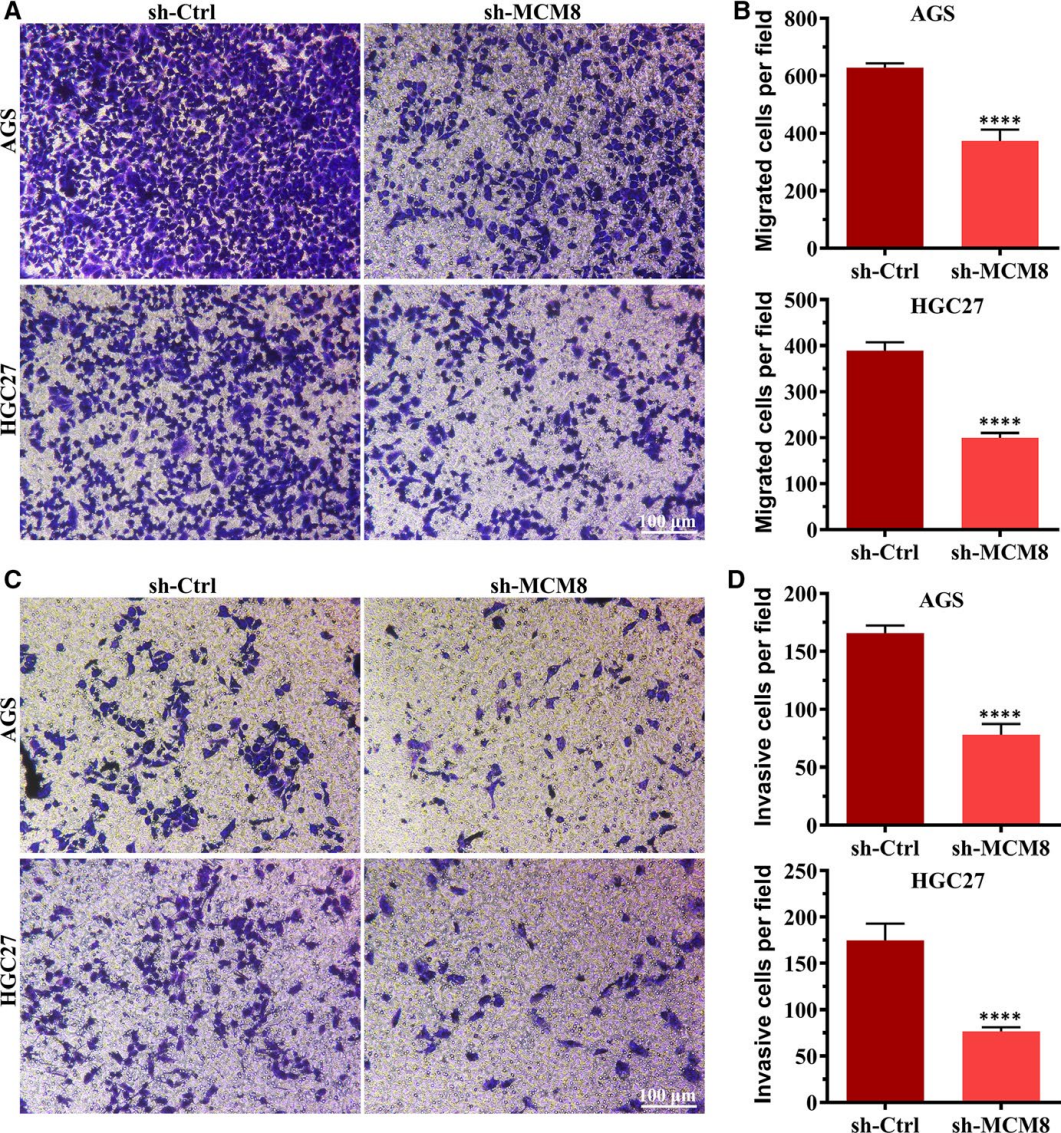
MCM8 knockdown inhibits cell metastasis. A, Representative images of the Transwell migration assay. Magnification: 100×. B, MCM8 knockdown suppresses cell migration. ****P < .001. C, Representative images of the Matrigel invasion assay. Magnification: 100×. D, MCM8 knockdown suppresses cell invasion. ****P < .001

To determine whether MCM8 affects cell metastasis, Transwell migration and Matrigel invasion assays were performed. As shown in Figure 6A, MCM knockdown reduced the number of migrated cells. The number of migrated cells was reduced from 628 ± 15 to 372 ± 40 (*P* < .0001) and from 389 ± 17 to 199 ± 10 (*P* < .0001) for AGS and HGC27, respectively (Figure 6B). Consistent with the results of the Transwell migration assay, the results of the Matrigel invasion assay showed that MCM8 knockdown significantly reduced the number of invasive cells (Figure 6C,D). These findings indicated that MCM8 promotes cell metastasis.

#### 3.5.2 MCM8 knockdown suppresses cell growth in a mouse xenograft model

**FIGURE 7:**
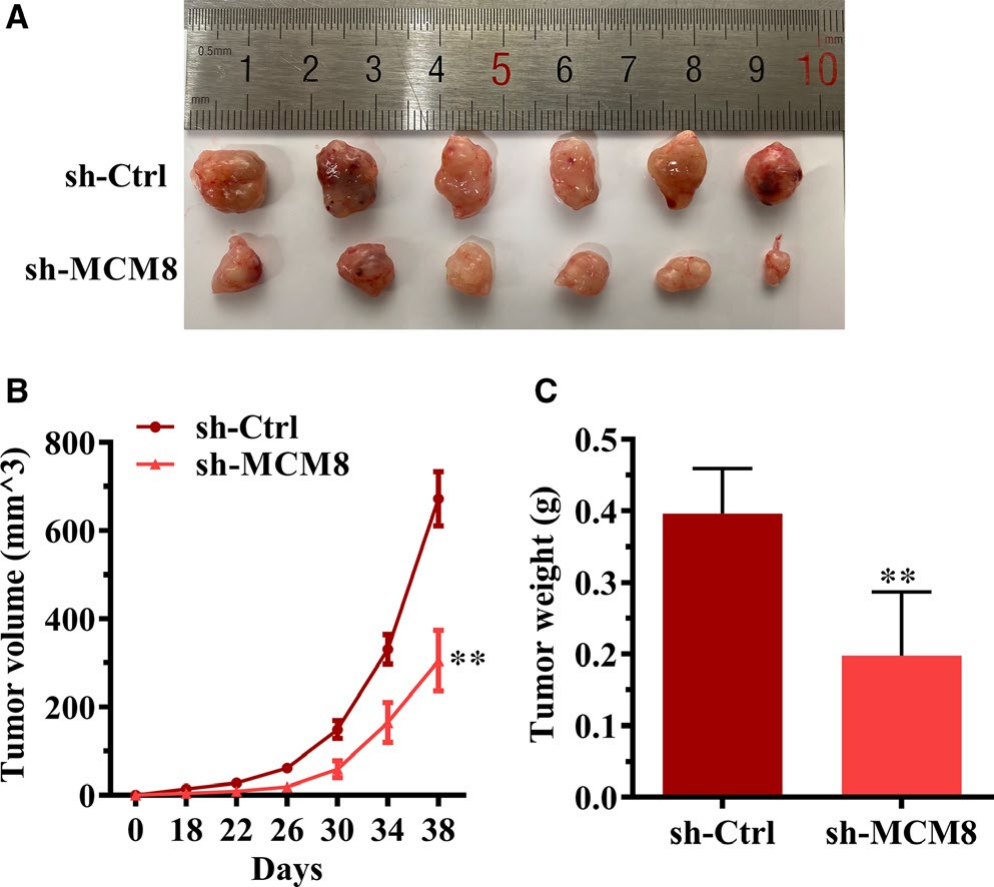
MCM8 knockdown suppresses tumorigenesis in vivo. A, Tumour images. In brief, 4 × 10^6^ HGC27 cells were subcutaneously injected into the right‐side dorsal flank of each mouse. The tumours were isolated on day 38. B, MCM8 knockdown significantly reduces the tumour volume. **P < .01. C, MCM8 knockdown reduces tumour weight. The tumour weight was recorded immediately after the tumours were harvested. **P < .01

To study the effect of MCM8 on cancer progression in vivo, HGC27 cells, with or without stable MCM8 knockdown, were subcutaneously injected into nude mice. Our results showed that MCM8 knockdown suppressed tumorigenesis (Figure 7A). The volume and weight of tumours were significantly reduced in the MCM8‐knockdown group when compared with those in the control group (Figure 7B,C).

## 4 DISCUSSION

In this study, we observed that several MCM proteins were up‐regulated in GC samples when compared with normal tissue samples according to data obtained from online databases. We further determined that overexpression of most MCMs was associated with poor prognosis based on the Kaplan‐Meier method, indicating their importance in tumour diagnosis and recurrence. One critical finding in this study was the significant overexpression of MCM8, not only in GC tissues but also in multiple cancer tissues when compared with normal tissues from the online GEPIA and Oncomine databases. The survival analysis based on online public databases revealed that higher MCM8 expression is associated with poor prognosis. These results are consistent with previous studies in lung adenocarcinoma and chronic myelogenous leukaemia^20^, ^21^, ^22^, ^23^, ^24^ and suggest possible roles of MCM8 in various cancers.

MCM8 is a new member of the MCM protein family, located contrapodal to GCD10 at chromosome band 20p12.3‐1.^31^ MCM8 acts as one of the DNA replication licensing factors, participating in the initiation and elongation of DNA replication.^32^, ^33^, ^34^ Furthermore, it is associated with chromosomal instability.^35^ Studies have reported that MCM8 can be recruited to the DNA repair site to promote DNA homologous recombination and double‐strand breaks.^14^, ^36^ Previous studies have indicated that DNA replication and DNA damage repair systems play important roles in inhibiting tumour proliferation through the preservation of genome integrity.^37^, ^38^ Recently, Cai et al have reported that the knockdown of MCM8 could reduce cell viability and induce apoptosis of chronic myelogenous leukaemia cells.^20^ In our study, we silenced the expression of MCM8 in two different GC cell lines (AGS and HGC27) using a lentivirus‐mediated shRNA and showed the inhibition of cell growth, as evidenced by reduced cell number, cell viability and the number of colonies. Moreover, MCM expression in association with the cell cycle reportedly controls DNA synthesis.^39^ Consistent with previous studies, we identified that MCM8 knockdown blocked G2/M progression in two GC cell lines, AGS and HGC27. Furthermore, the mitochondrial pathway is a crucial apoptotic pathway. The anti‐apoptotic protein, Bcl‐2, and the pro‐apoptotic protein, Bax, both belonging to the Bcl‐2 family, are critical regulators of this pathway.^40^, ^41^ In this study, we observed that MCM8 knockdown increased the apoptosis in both AGS and HGC27 cells. The protein expression level showed that the ratio of Bax/Bcl‐2 in AGS cells increased following MCM8 knockdown. These findings demonstrated that disrupting the balance of proliferation/apoptosis could be attributed to MCM8 overexpression in GC development. However, the precise underlying mechanism requires further investigation.

In summary, we observed MCM8 overexpression in GC tissues and demonstrated a correlation between MCM8 up‐regulation and poor patient survival. MCM8 knockdown exerted anti‐tumour activity both in vitro and in vivo. These findings indicate the biological function of MCM8 in GC and suggest that MCM8 could be used as a potential biomarker for this cancer.

## CONFLICT OF INTEREST

The authors declare that they have no competing interests.

## AUTHOR CONTRIBUTIONS

**Bin Huang:** Conceptualization (lead); Data curation (lead); Formal analysis (lead); Funding acquisition (equal); Investigation (lead); Methodology (lead); Project administration (lead); Resources (lead); Software (lead); Supervision (lead); Validation (lead); Visualization (lead); Writing‐original draft (lead); Writing‐review & editing (lead). **Minghe Lin:** Conceptualization (equal); Data curation (supporting); Formal analysis (supporting); Investigation (supporting); Methodology (supporting); Project administration (equal); Resources (supporting); Software (supporting); Supervision (supporting); Validation (supporting); Visualization (equal); Writing‐original draft (supporting); Writing‐review & editing (equal). **Lisha LU:** Conceptualization (equal); Data curation (equal); Investigation (equal); Methodology (equal); Project administration (equal); Resources (supporting); Writing‐original draft (supporting); Writing‐review & editing (supporting). **Wujin Chen:** Conceptualization (supporting); Formal analysis (supporting); Funding acquisition (supporting); Project administration (supporting); Resources (lead); Validation (supporting); Visualization (equal). **JingZhuang Tan:** Conceptualization (supporting); Data curation (supporting); Formal analysis (supporting); Validation (supporting). **Jinyan Zhao:** Conceptualization (supporting); Data curation (supporting); Formal analysis (supporting); Funding acquisition (supporting); Investigation (supporting); Methodology (supporting); Project administration (supporting); Resources (supporting). **Zhiyun Cao:** Project administration (supporting). **Xiaoqin Zhu:** Writing‐original draft (supporting); Writing‐review & editing (supporting). **Jiu‐Mao Lin:** Conceptualization (lead); Data curation (supporting); Formal analysis (supporting); Funding acquisition (lead); Investigation (supporting); Methodology (supporting); Project administration (supporting); Resources (lead); Supervision (lead).

## Supporting information

Fig S1

Table S1

## Data Availability

The data that support the findings of this study are available from the corresponding author upon reasonable request.
