# Supplementary figures and images for: Identification of mini‐chromosome maintenance 8 as a potential prognostic marker and its effects on proliferation and apoptosis in gastric cancer
Source: J Cell Mol Med. 2020 Nov 6;24(24):14415–25. doi: 10.1111/jcmm.16062 (PMC7753872; doi:10.1111/jcmm.16062)

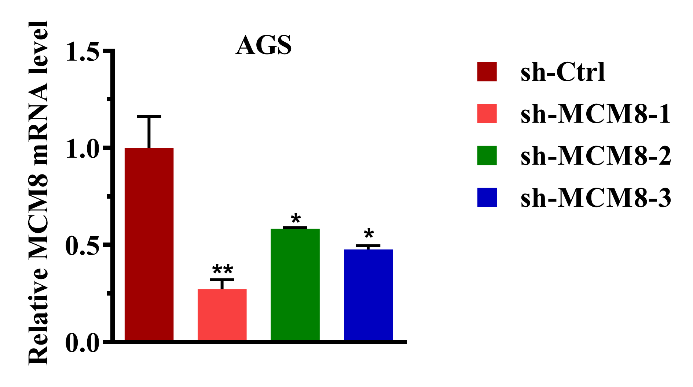

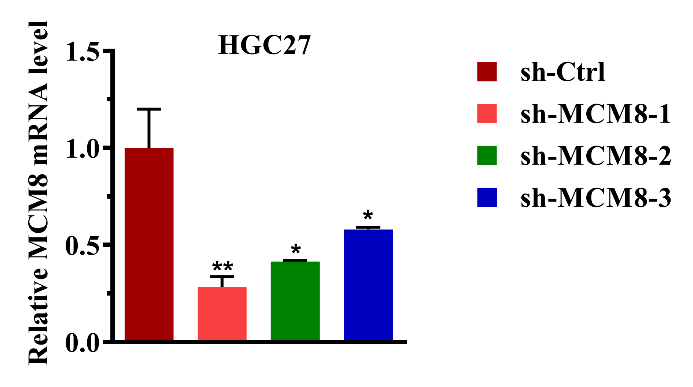


**Supplemental Fig. 1. The knockdown-efficiencies of shRNAs were determined using RT-qPCR.**

Supplement: Supplementary file 1 — Fig S1 [file JCMM-24-14415-s001.docx]
