# Supplementary material for: Identification of mini‐chromosome maintenance 8 as a potential prognostic marker and its effects on proliferation and apoptosis in gastric cancer
Source: J Cell Mol Med. 2020 Nov 6;24(24):14415–25. doi: 10.1111/jcmm.16062 (PMC7753872; doi:10.1111/jcmm.16062)
Supplement: Supplementary file 2 — Table S1 [file JCMM-24-14415-s002.docx]

**Supplemental table 1. The targeting sequence of the shRNAs and primers for plasmid construction**

| sh-Ctrl-targeting-sequence | 5′-TTCTCCGAACGTGTCACGTAA-3′ |
| --- | --- |
| sh-Ctrl-F | 5′-GATCCGTTCTCCGAACGTGTCACGTAATTCAAGAG  ATTACGTGACACGTTCGGAGAATTTTTTC-3′ |
| sh-Ctrl-R | 5′-AATTGAAAAAATTCTCCGAACGTGTCACGTAATCT  CTTGAATTACGTGACACGTTCGGAGAACG-3′ |
| sh-MCM8-1- targeting sequence | 5′-TAGCTCTCCTTTGATTGAGAAGATT-3′ |
| sh-MCM8-1-F | 5′-GATCCGTAGCTCTCCTTTGATTGAGAAGATTCTC  GAGAATCTTCTCAATCAAAGGAGAGCTATTTTTTG-3′ |
| sh-MCM8-1-R | 5′- AATTCAAAAAATAGCTCTCCTTTGATTGAGAAGA  TTCTCGAGAATCTTCTCAATCAAAGGAGAGCTACG-3′ |
| sh-MCM8-2- targeting sequence | 5′-CATGGAGCAGCAAAGTATTAGTCTT-3′ |
| sh-MCM8-2-F | 5′-GATCCGCATGGAGCAGCAAAGTATTAGTCTTCTC  GAGAAGACTAATACTTTGCTGCTCCATGTTTTTTG-3′ |
| sh-MCM8-2-R | 5′-AATTCAAAAAACATGGAGCAGCAAAGTATTAGTC  TTCTCGAGAAGACTAATACTTTGCTGCTCCATGCG-3′ |
| sh-MCM8-3- targeting sequence | 5′-CCCTGGTACTTGGTGATCAAGGTAT-3′ |
| sh-MCM8-3-F | 5′-GATCCGCCCTGGTACTTGGTGATCAAGGTATTTCAA  GAGAATACCTTGATCACCAAGTACCAGGGTTTTTTG-3′ |
| sh-MCM8-3-R | 5′-AATTCAAAAAACCCTGGTACTTGGTGATCAAGGTAT  TCTCTTGAAATACCTTGATCACCAAGTACCAGGGCG-3′ |
